# Responses of Low-Cost Input Combinations on the Microbial Structure of the Maize Rhizosphere for Greenhouse Gas Mitigation and Plant Biomass Production

**DOI:** 10.3389/fpls.2021.683658

**Published:** 2021-06-30

**Authors:** Caio Augusto Yoshiura, Andressa Monteiro Venturini, Lucas Palma Perez Braga, Aline Giovana da França, Maria do Carmo Catanho Pereira de Lyra, Siu Mui Tsai, Jorge Luiz Mazza Rodrigues

**Affiliations:** ^1^Cell and Molecular Biology Laboratory, Center for Nuclear Energy in Agriculture, University of São Paulo, Piracicaba, Brazil; ^2^Genome Laboratory, Agronomic Institute of Pernambuco, Recife, Brazil; ^3^Soil EcoGenomics Laboratory, University of California, Davis, Davis, CA, United States

**Keywords:** microbial ecology, denitrification, mesocosm, plant growth-promoting rhizobacteria, methanotrophy, methanogenesis

## Abstract

The microbial composition of the rhizosphere and greenhouse gas (GHG) emissions under the most common input combinations in maize (*Zea mays* L.) cultivated in Brazil have not been characterized yet. In this study, we evaluated the influence of maize stover coverage (S), urea-topdressing fertilization (F), and the microbial inoculant *Azospirillum brasilense* (I) on soil GHG emissions and rhizosphere microbial communities during maize development. We conducted a greenhouse experiment and measured methane (CH_4_), carbon dioxide (CO_2_), and nitrous oxide (N_2_O) fluxes from soil cultivated with maize plants under factorial combinations of the inputs and a control treatment (F, I, S, FI, FS, IS, FIS, and control). Plant biomass was evaluated, and rhizosphere soil samples were collected at V5 and V15 stages and DNA was extracted. The abundance of functional genes (*mcrA*, *pmoA*, *nifH*, and *nosZ*) was determined by quantitative PCR (qPCR) and the structure of the microbial community was assessed through 16S rRNA amplicon sequencing. Our results corroborate with previous studies which used fewer input combinations and revealed different responses for the following three inputs: F increased N_2_O emissions around 1 week after application; I tended to reduce CH_4_ and CO_2_ emissions, acting as a plant growth stimulator through phytohormones; S showed an increment for CO_2_ emissions by increasing carbon-use efficiency. IS and FIS treatments presented significant gains in biomass that could be related to Actinobacteria (19.0%) and Bacilli (10.0%) in IS, and Bacilli (9.7%) in FIS, which are the microbial taxa commonly associated with lignocellulose degradation. Comparing all factors, the IS (inoculant + maize stover) treatment was considered the best option for plant biomass production and GHG mitigation since FIS provides small gains toward the management effort of F application.

## Introduction

Several inputs have been studied and documented to improve crop development and production, increasing soil health and plant resistance to pathogens and seasonal variations (Thangarajan et al., 2013; Wang et al., 2016; Abbott et al., 2018; Sharma and Bali, 2018). However, the excessive and indiscriminate use of synthetic nitrogen fertilizers can cause an imbalance in the nitrogen cycle at huge economic costs. These are associated with human health problems due to drinking water contamination by high nitrate concentrations and environmental problems, such as freshwater eutrophication and climate change by the increase of nitrous oxide (N_2_O) and carbon dioxide (CO_2_) emissions (Galloway et al., 2008; Ward, 2009; Zamanian et al., 2018), enforcing the continuous search for alternatives to improve food production. Currently, three inputs – synthetic nitrogen fertilizers, commercial microbial inoculants, and plant residues coverage from the adoption of no-tillage practices – have been commonly used in agricultural practices to increase crop yield and production. Microbial inoculants, such as plant-growth-promoting rhizobacteria (PGPR) are alternatives to nitrogen fertilization and are commonly employed as biofertilizers, phytostimulators, and biocontrollers (Okon and Labandera-Gonzalez, 1994; Bloemberg and Lugtenberg, 2001). On the other hand, no-tillage practices have been increasing among crop management systems around the globe since they provide plant residues for soil coverage and nutrient supply (Busari et al., 2015; Pittelkow et al., 2015) and improve the physicochemical characteristics and carbon storage of the soil by increasing the CO_2_ sequestration (Rigon and Calonego, 2020). For instance, no-tillage areas in Brazil, the United States, and China are implemented in more than 50% of their total crop areas (He et al., 2010; USDA, 2010; Freitas and Landers, 2014).

Brazil is the third worldwide producer of maize (*Z. mays* L.), the second most important crop for the country, whose production increased more than 6% through the last 10 years (CONAB, 2019). Recently, studies on the evaluation of maize managements are focused on crop yield, soil greenhouse gas (GHG) emissions (Calvo et al., 2016; Cambouris et al., 2016; Müller et al., 2016), or the characterization of bulk and rhizosphere soil microbial communities using molecular approaches (Peiffer et al., 2013; Li et al., 2014). These studies about crop management are driven mostly by the increasing demand for food to feed human populations (FAO, 2009) and global climate changes due to the increase of GHG emissions, which are responsible for extreme weather events and rising sea levels, among other environmental impacts, in addition to threatening wildlife, as reported by the IPCC (2014).^1^ However, few studies have characterized the microbial composition of the maize rhizosphere, aiming to prospect for potential mitigators of GHG emissions, and test the combined effects of agricultural inputs commonly used in Brazil (Teixeira et al., 2019), thus evaluating combinations for balanced and sustainable managements with less GHG emissions and higher maize production. In Brazil, around 35.7 Gg of N_2_O [i.e., 10.6 Tg in carbon dioxide equivalent (CO_2_ equivalent)] was emitted in 2010 from nitrogen fertilization (Brasil, 2016). Aiming at N_2_O mitigation, recent technologies have been developed to reduce losses from fertilizers by volatilization and leaching, such as fertilizer coating or the addition of urease inhibitors (Barbarena et al., 2019; Bortoletto-Santos et al., 2019). The losses from fertilizers are closely related to the conventional urea that represents about 50% of the total N applied in Brazilian agriculture in the last two decades due to its low cost (Santos et al., 2020). Although the stabilized and slow-release urea is an alternative to reduced nitrogen losses by volatilization, it did not increase the nitrogen content in plants compared to conventional urea (Cancellier et al., 2016). Nevertheless, information about nitrogen fertilization remains incomplete and more than ever, results are needed to help establish sustainable management to reduce N_2_O without compromising crop yields (Abalos et al., 2016).

Microbial processes in the rhizosphere affect plant growth (Bonkowski et al., 2000), provide protection against pathogens and environmental stress (Liu et al., 2020; Tkacz and Poole, 2021), and are responsible for relevant activities related to biogeochemical cycles (Li et al., 2014). Roots modify soil properties by releasing several low-molecular-mass compounds, polymerized sugars, root border cells, and dead root cap cells that alter the structure, functions, and interactions of the microbial populations (Philippot et al., 2013). Besides these, the incorporation of inputs also modifies the soil quality and nutrient content, feeding plants and microbial populations (Wieland et al., 2001; Geisseler and Scow, 2014). Therefore, bioaugmentation using PGPR, such as *Azospirillum brasilense*, could act as diazotrophic bacteria for biological nitrogen (N_2_) fixation (Hartmann and Burris, 1987; Somers et al., 2005; Bashan et al., 2014; Backer et al., 2018) and promote other activities, such as phosphate solubilization, degradation of siderophores, and biological control of soil-borne pathogens (Bashan et al., 2004).

Toward the problem of the excessive use of urea as a nitrogen fertilizer in Brazilian fields, our hypothesis was to evaluate the necessity of urea-topdressing fertilization to increase plant biomass for high crop yield since the use of microbial inoculants and no-tillage practices are beneficial alternatives. In this study, we evaluated the influence of maize stover coverage, urea-topdressing fertilizer, and the microbial inoculant, *A. brasilense* on soil GHG emissions and the microbial composition of the maize rhizosphere during plant development through a mesocosm experiment. Hereby, we established direct comparisons among all factorial treatments, covering knowledge gaps related to the effects of these input combinations on soil GHG fluxes and microbial communities.

## Materials and Methods

### Experimental Design and Setup

**Figure 1:**
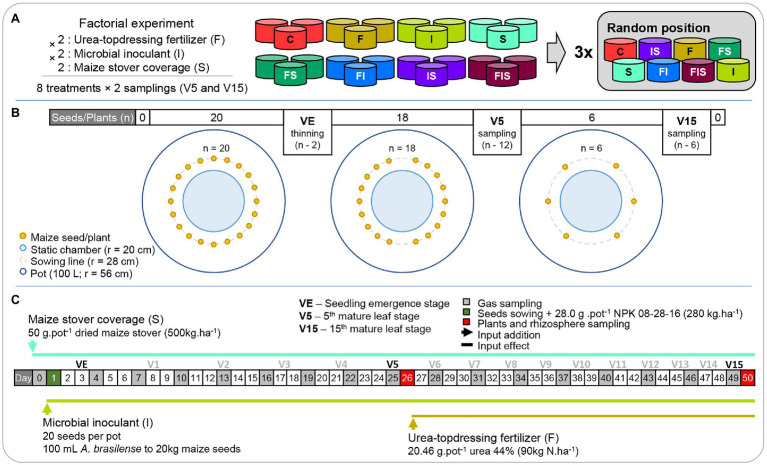
Schemes of the **(A)** experimental design and **(B)** mesocosm pots through the **(C)** timeline. Treatments: C, Control; F, Urea-topdressing fertilization at V5; I, *A. brasilense* inoculant on seeds sowing; FI, F + I; S, Maize stover coverage; FS, F + S; IS, I + S; FIS, F + I + S.

A factorial mesocosm experiment (Figure 1) was assembled to test the influence of maize stover, urea-topdressing fertilization, and microbial inoculant (*A. brasilense*) – 2 (with and without urea-topdressing fertilization) × 2 (with and without microbial inoculant) × 2 (with and without maize stover) × 2 (V5 and V15 sampling times; Figure 1A) – on the microbial composition of the maize rhizosphere, maize biomass gains, and GHG emissions. The factorial design resulted in eight treatments [urea-topdressing fertilization (F), microbial inoculant (I), maize stover coverage (S), and the combinations F + I (FI), F + S (FS), I + S (IS), F + I + S (FIS), and a control treatment (C)] with the following two sampling times: the next morning after the observation of the 5th mature leaf (V5) and maize stage of the 15th mature leaf (V15). All treatments were established in three replicates, in which each replicate was placed randomly in one block, that is, the experiment totalized three blocks containing eight pots each.

The mesocosm experiment was assembled in a greenhouse at the University of São Paulo – Center for Nuclear Energy in Agriculture (USP-CENA) in the maize second-crop season (Summer–Fall) of 2016. The topsoil (from 0 to 25 cm) from about 30 m^2^ was obtained from an agricultural field at the Anhumas Experimental Station (22°50′28.22″S, 48°1′1.7″W). Twenty-four pots of 100 L (with 8 cm of gravel in the bottom layer for water drainage) were filled with the homogenized topsoil. The soil was classified as a dystrophic red-yellow latosol (IBGE, 2018) – oxisol, according to the US Soil Taxonomy (Soil Survey Staff, 1999), or Ferralsols, in the International Soil Classification System (IUSS Working Group WRB, 2015); and it was selected on the basis that dystrophic latosols represent a major part of soils used for maize production in Brazil (CONAB, 2018; IBGE, 2018). After sampling, aliquots (approximately 300 g) were sent to the Department of Soils of the University of São Paulo – Luiz de Queiroz College of Agriculture (USP-ESALQ) for physicochemical analysis, established for tropical soils according to the Agronomic Institute of Campinas (IAC; van Raij et al., 2001), to calculate the correction of soil fertility. Therefore, 28 g per pot (280 kg ha^−1^) of a 08-28-16 NPK formulation was added to the seed furrows during sowing, according to the fertility recommendation (productivity more than 6 t ha^−1^; EMBRAPA, 2015) for plant health maintenance until the V15 sampling.

Maize plants were grown using seeds with VT Pro Yieldgard technology (AG 8088; Sementes Agroceres – Monsanto, Brazil), suitable for grain and silage production on the first and second crops with high-temperature tolerance. In each pot, 20 seeds were sowed at 9.5 cm of distance in a circular line (*r* = 28 cm; Figure 1B). After seedling emergence (VE), 18 seedlings were kept until the V5 sampling time. At V5, 12 plants were collected, the remaining six for the V15 sampling time. Therefore, the experiment design was planned to simulate the planting population rates of 60,000 plants per hectare at V15.

The inputs were added to the experiment as described in the timeline (Figure 1C). Dried maize stover was collected from the Anhumas Experimental Station and applied in a proportion of 50 g per pot, simulating in-field conditions of maize residue coverage (500 kg ha^−1^; S treatments). The strains, AbV5 and AbV6 of *A. brasilense* commercialized with a minimum concentration of 2 × 10^8^ UFC ml^−1^ by Stoller do Brasil LTDA (Brazil) as the liquid inoculant Masterfix L Gramíneas was sprayed at 1 ml per 200 g of seeds (100 ml per hectare is equivalent to 100 ml per 20 kg of seeds) following the instructions of the manufacturer (I treatments). In non-inoculated treatments, 1 ml of sterilized water was sprayed instead. Urea-topdressing fertilization was applied after the V5 sampling time according to the field management conditions for a high yield of maize (90 kg N ha^−1^), representing 20.46 g of urea (44% N) per pot (F treatments; EMBRAPA, 2015).

The different times of the application of the inputs implied different activation times of their effects. The maize stover coverage and *A. brasilense* inoculant inputs started their influence at the beginning of the experiment, and the urea-topdressing fertilization started its influence after V5. This means that F treatments (F, FI, FS, and FIS) have the same characteristics as C, I, S, and IS, respectively before V5 treatments.

The experiment was conducted for 50 days until the V15 stage of the development of the maize plants. During the experiment, soil moisture was kept similar among treatments with daily irrigation – 1 L at initial stages (VE stage and after) and up to 8 L before reproductive stages (before V15) per pot – based on maize water consumption, considering each phenological phase, treatment, and weather (EMBRAPA, 2015). Soil temperature oscillated from 21 to 30°C, and the air temperature inside the greenhouse varied from 21 to 45°C, at daylight hours, during the experimental period.

### Gas Sampling and Analyses

Static round-chambers (20-cm inner diameter) were installed in the center of each pot to monitor the differences in gas fluxes during the growth of maize plants until V15. The anchors of the chambers were placed at 5 cm depth from the surface so as not to affect the soil water movement and to ensure the retention of gases in the chambers (Cerri et al., 2013).

Gas samplings were carried out in the morning between 10 a.m. and 12 p.m. (BRT; UTC-3:00) before the beginning of the experiment (Day 0) and at every 72 h after seed sowing until V15 (Days 4, 7, 10, 13, 16, 19, 22, 25, 28, 31, 34, 37, 40, 43, 46, and 49; Figure 1B). After attaching the chamber lids on their anchors, gas samples of 20 ml were collected for 45 min (1, 15, 30, and 45 min; i.e., T1, T15, T30, and T45, respectively) from each chamber in plastic syringes to be read (after the gas sampling) in the SRI 8610C gas chromatography instrument (SRI Instruments, United States) that was set to detect methane (CH_4_) and CO_2_ by a flame ionization detector (FID), and N_2_O gas by an electron capture detector (ECD), according to the operating manual of the manufacturer. In addition, a sample of the ambient air at the initial time (T0) of each sampling day was collected as quality control for gas measurement normalization, and air temperature data were collected for gas law correction (Cerri et al., 2013).

Greenhouse gas emissions were evaluated from 1 day before sowing seeds until V15 (50 days) of the development of the maize plants. The emissions of CH_4_, CO_2_, and N_2_O were determined by the mean of total cumulative fluxes from the same treatment, obtained through the linear projection of emission times (T1–T45) from each sampling day and the sum of the results obtained throughout the experimental period (Day 0–49). The values of CH_4_ and N_2_O were transformed into carbon dioxide equivalent (CO_2_ equivalent in g pot^−1^) by multiplying with 34 and 298, respectively, based on the 100-year global warming potential (GWP_100_) with climate-carbon feedbacks (IPCC, 2013).

### Rhizosphere Soil Sampling and Biomass of Maize Plants

The entire maize plants – V5 (12 plants) and V15 (6 plants) – from each pot were harvested, totalizing 48 samples during the experiment. After sampling, the excess of soil on the plant roots (40 cm length) was removed by shaking, and the soil firmly attached (moistened) by exudates was considered as the rhizosphere. Rhizosphere soil samples from each plant were collected, homogenized, conditioned in 15 ml tubes, fast-frozen using liquid nitrogen (N_2_), and stored at −80°C for further analysis. Soil samples were also collected to check physicochemical characteristics at V15 (Supplementary Table 1; Supplementary Figure 1). Shoots of plants harvested were dried in the oven at 60°C for 5 days and weighted to evaluate the development of plants and biomass gains during the experiment.

### Genomic Analysis

For molecular analysis, DNA extraction from 0.5 g of each rhizosphere soil sample was carried out using DNeasyPowerSoil Kit (Qiagen, CA, United States), following the protocol of the manufacturer. The concentration and quality of the DNA samples were evaluated on 1% GelRed-stained agarose gels (electrophoresis conditions of 80 V by 40 min) in sodium boric acid buffer (Brody and Kern, 2004) and on a Nanodrop 2000 c spectrophotometer (Thermo Fisher Scientific, MA, United States). The DNA samples were stored at −20°C for molecular analysis.

#### Quantitative PCR

Generalist primer sets for functional microbial groups were used in qPCR assays. Primers related to CH_4_ and N_2_O production and consumption and nitrogen fixation were retrieved from the literature. However, primers associated with the N_2_O production – nitric oxide reductase genes (*norB* and *P450nor*) – remained unsatisfactory for a wide range of taxonomic groups (Ma et al., 2019) and were not used in this study. Thus, primers targeting particulate methane monooxygenase (*pmoA*) and methyl-coenzyme M reductase (*mcrA*) genes were selected to evaluate the consumption and production of CH_4_, respectively. Primers targeting nitrous oxide reductase (*nosZ*) and nitrogenase iron protein (*nifH*) genes were selected to evaluate the denitrification of N_2_O to dinitrogen (N_2_) and the atmospheric N_2_ fixation, respectively.

Standard curves for absolute quantification were prepared from serial dilutions containing between 10^5^ and 10^0^ copies of the target genes, obtained from strains of the German Collection of Microorganisms and Cell Cultures (DSMZ, Germany): *nifH* from *Bradyrhizobium japonicum* (DSMZ 30131), *nosZ* from *Paraburkholderia phymatum* (DSMZ 17167), *mcrA* from *Methanolinea mesofila* (DSMZ 23604), and *pmoA* from *Methylosinus sporium* (DSMZ 17706).

Quantitative PCR assays from the samples of V5 and V15 were carried out, under modified thermal cycling conditions (Supplementary Table 2), in triplicates containing 1X Maxima SYBR Green/ROX qPCR Master Mix (2X; Thermo Fisher Scientific, Vilnius, Lithuania), 1.0 μm of each universal primer (Supplementary Table 2), 10 ng of DNA, and ultrapure deionized water to complete 10 μl. All qPCR assays were carried out in a StepOnePlus Real-Time PCR System instrument (Applied Biosystems, MA, United States) and analyzed using the StepOne Software v2.3, reaching efficiency between 90 and 100% and 0.99 of pipetting error. Gene abundance comparisons were performed relative to each other using the initial amount of sample DNA as a normalization parameter among treatments and considered in the calculations.

#### 16S rRNA Amplicon Sequencing

The taxonomic composition of microbial communities influenced by the treatments at V15 (24 samples) was investigated by 16S rRNA amplicon sequencing. For this, PCRs were carried out containing 1X Phusion Hot Start II High-Fidelity PCR Master Mix (2X; Thermo Fisher Scientific, MA, United States), 0.5 μm of each primer set (forward + reverse for each sample, as described in Supplementary Table 3) for the V4 region from the Earth Microbiome Project (EMP),^2^ 1 μl of DNA, and ultrapure deionized water to complete 20 μl. The thermal cycling conditions were 30 s of denaturation at 98°C, followed by 27 rounds of temperature cycling (98°C for 30 s, 50°C for 30 s, and 72°C for 20 s) and a final extension at 72°C for 7 min. All reactions were carried out in a C1000 Touch™ Thermal Cycler with Dual 48/48 Fast Reaction Module (Bio-Rad, CA, United States). Aliquots (5 μl) of the PCR products were checked on 1% GelRed-stained agarose gels running at 80 V for 40 min and quantified using Qubit 2.0 Fluorometer (Invitrogen, MA, United States) following the instructions of the manufacturer. All PCR products were purified using QIAquick PCR Purification Kit (Qiagen, Hilden, Germany) following the instructions of the manufacturer. The purified PCR products were sent to the Genome Center Facility of the University of California Davis (Davis, CA, United States) for paired-end amplicon sequencing (2 × 250 bp) in a HiSeq 2500 platform (Illumina Inc., CA, United States).

### Computational and Statistical Analysis

The gas fluxes were analyzed by Shapiro–Wilk and Kolmogorov–Smirnov normality tests and Levene’s homogeneity test in order to define the most appropriate statistical test to be used to evaluate differences among treatments. Two-way analysis of variance (ANOVA) followed by the Tukey Honest Significant Difference (HSD) *post-hoc* test for multiple comparisons at *p* < 0.05 were performed using the agricolae package v1.2-8 (Mendiburu, 2017). For non-parametric data, Kruskal–Wallis with the *post-hoc* Dunn’s test from the dunn.test package v1.3.5 (Dinno, 2017) was implemented, all in R-statistical environment (version 3.4.3.; R Core Team, 2017).

Comparisons of plant biomass, soil physicochemical properties, and gene abundance were also performed by two-way ANOVA, as previously described.

Raw nucleotide sequences of 16S rRNA amplicons were analyzed using Qiime2 microbiome bioinformatics platform version 2018.8 (Bolyen et al., 2019). Sequences were treated using dada2 version 2017.6.0 (sequences were maintained if *Q* > 30, truncated to 175 bp, and chimeric filtered; Callahan et al., 2016), then rarefied to 50,000 sequences and aligned to Silva 132 release database (Quast et al., 2013) based on 99% sequence identity as the taxonomic reference. Raw sequences were deposited in the SRA database (accession number PRJNA495686).

Diversity indices were calculated from the aligned sequences at the order level using Qiime2. Non-metric multidimensional scaling (NMDS) analysis (Bray-Curtis) and phylogenetic (weighted UniFrac) were generated using vegan v2.4-6 (Oksanen et al., 2017) and ape v5.0 (Paradis et al., 2004) packages and plotted using ggplot2 v2.2.1 (Wickham, 2009) in R-statistical environment (R Core Team, 2017).

STAMP v2.1.3 (Parks et al., 2014), a graphical software for statistical analysis of taxonomical and functional profiles, was used to determine statistical differences among rhizosphere-soil treatments. The values of *p* were calculated using Welch’s *t*-test two-sided with Welch’s inverted method to calculate confidence intervals at 95%. The Storey False Discovery Rate (FDR) multiple test correction was applied (*p* < 0.05) with an effect size filter of difference between proportions (*DP* < 1.00).

## Results

### Gas Emissions

**Figure 2:**
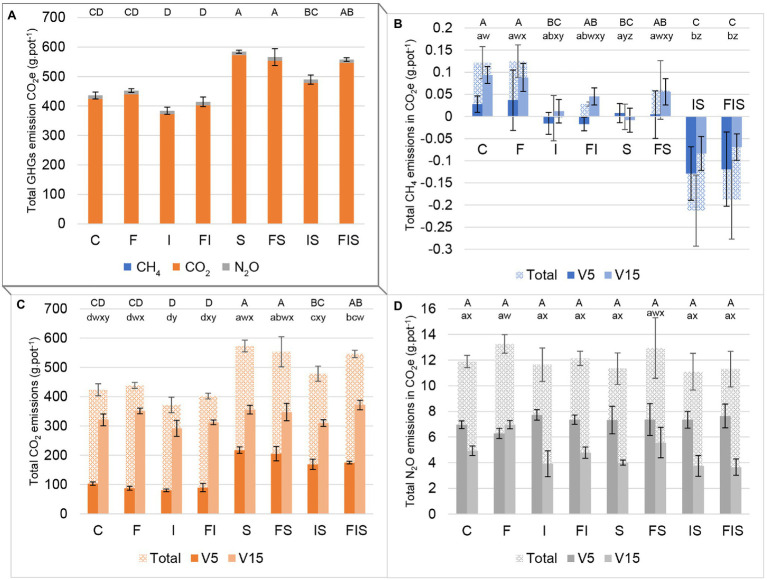
Emissions in carbon dioxide equivalent (CO_2_ equivalent) from the maize soil-rhizosphere experiment: **(A)** sum of all three main GHGs, **(B)** methane (CH_4_), **(C)** carbon dioxide (CO_2_), and **(D)** nitrous oxide (N_2_O). Bars with the same letter are not significantly different (*p* < 0.05). Three groups of series of letters compare treatments: uppercase letters (ABCD) on the top of bars compare differences among total emissions of the entire experiment; lowercase letters close to bars compare each stage period (*abcd* for V5 and *wxyz* for V15). V5 includes accumulated gas measurements before the rhizosphere sampling at V5, while V15 includes accumulated gas measurements between both rhizosphere sampling times (V5 and V15). Total bars are the sum of both periods (V5 and V15). Treatments: C, Control; F, Urea-topdressing fertilization at V5; I, *A. brasilense* inoculant on seeds sowing; FI, F + I; S, Maize stover coverage; FS, F + S; IS, I + S; FIS, F + I + S.

As a reference, total GHG emissions in the control group were equivalent to 435.79 ± 11.73 g pot^−1^ in CO_2_ equivalent (Figure 2A). Carbon dioxide fluxes from soils were higher than N_2_O and CH_4_ fluxes in all treatments.

Considering the total sum of GHG emissions (Figure 2A), treatments containing *A. brasilense* (I/FI/IS/FIS) presented, respectively, with a reduction of 11.9, 8.4, 16.2, and 1.6% in emissions compared to its “control treatment” without the inoculant (C, F, S, and FS, respectively), with the IS treatment demonstrating the most pronounced effect of GHG mitigation (*p* < 0.05). The GHG emissions obtained from the urea-topdressing fertilization treatments compared to the other groups (i.e., F:C, FI:I, FS:S, and FIS:IS) varied 3.8, 7.9, −3.1%, and 13.8%, respectively. Finally, the treatments influenced by stover (i.e., S, FS, IS, and FIS), strongly increased the GHG emissions by 34.1, 25.2, 27.5, and 34.5% in comparison to the other treatments (i.e., C, F, I, and FI).

Analyzing individually (Figures 2B–D), treatments, I and FI presented a tendency to reduce CH_4_ emissions before V5, while the S treatment presented the same tendency after V5. This tendency of reduction of CH_4_ from I, FI, and S treatments was potentialized and become significant when combined in IS and FIS treatments, before and after V5 (Figure 2B).

The CO_2_ measurements (Figure 2C) showed that S treatments (i.e., S, FS, IS, and FIS) are related to increments in gas emissions until V5. However, I treatments except for FIS (i.e., I, FI, and IS) presented reduced emissions after V5 in comparison to the other treatments.

Comparisons between F treatments and their “control treatment” (i.e., F:C, FI:I, FS:S, and FIS:IS) after V5, urea-topdressing fertilizer significantly increased by 41.4% N_2_O emissions (Figure 2D) in F:C comparison (*p* < 0.01) and showed a slight increment of 39.2% in FS:S comparison. However, increment differences in FI:I and FIS:IS comparisons were not significant, with 21.9 and 2.5%, respectively.

### Maize Plant Biomass

**Figure 3:**
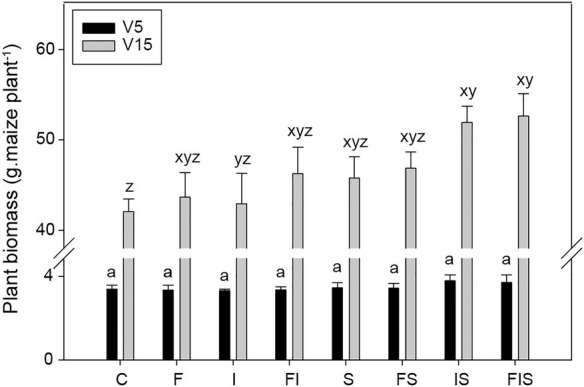
Biomass average of maize plants collected at V5 (*n* = 12) and V15 (*n* = 6). Bars with the same letter are not significantly different (*p* < 0.05). Two groups of series of letters compare treatments from distinctly stage periods (*a* for V5 and *xyz* for V15). Treatments: C, Control; F, Urea-topdressing fertilization at V5; I, *A. brasilense* inoculant on seeds sowing; FI, F + I; S, Maize stover coverage; FS, F + S; IS, I + S; FIS, F + I + S.

At V5, maize plants from IS and FIS treatments (identical treatments before the urea-topdressing fertilization) showed a tendency for the highest gains in plant biomass – i.e., highest individual plant biomass gain. However, the average increases were not different from others (*n* = 12), probably due to the influence of the initial fertility correction. This tendency of plant biomass gains raised at V15, and both IS and FIS treatments presented a significant increase in biomass (*n* = 6; *p* < 0.05), with 23.4 and 25.2% more than C condition, respectively (Figure 3). However, they were considered similar to the other treatments.

### Quantitative PCR

At V5, the average number of copies per gram of soil (copies. g soil^-1-1^) ranged from 6.64 × 10^3^ to 5.52 × 10^4^ for the *mcrA* gene; from 3.28 × 10^3^ to 6.38 × 10^3^ for *pmoA*; from 9.07 × 10^4^ to 2.60 × 10^5^ for *nifH*; and from 3.26 × 10^4^ to 2.48 × 10^5^ for *nosZ*. In comparison, the quantification of V15 samples ranged from 5.67 × 10^2^ to 2.04 × 10^3^ for the *mcrA* gene; from 3.35 × 10^3^ to 9.50 × 10^3^ for *pmoA*; from 1.67 × 10^3^ to 1.32 × 10^5^ for *nifH*; and from 4.81 × 10^4^ to 1.75 × 10^5^ for *nosZ* (Supplementary Figure 2).

In general, the S treatments (S, FS, IS, and FIS) contained more copies of the *mcrA* gene (above 4.00 × 10^4^copies.g soil^−1^) than the other treatments at V5, and *pmoA* (above 7.00 × 10^3^copies.g soil^−1^) at V15. However, all treatments presented a small number of copies (up to 2.04 × 10^3^copies.g soil^−1^) of *mcrA* at V15.

Nevertheless, differences with undistinguishable patterns between the sampling times (V5 and V15) among treatments were observed for all genes (Supplementary Table 2).

### Alpha and Beta Diversity Analyses

**Figure 4:**
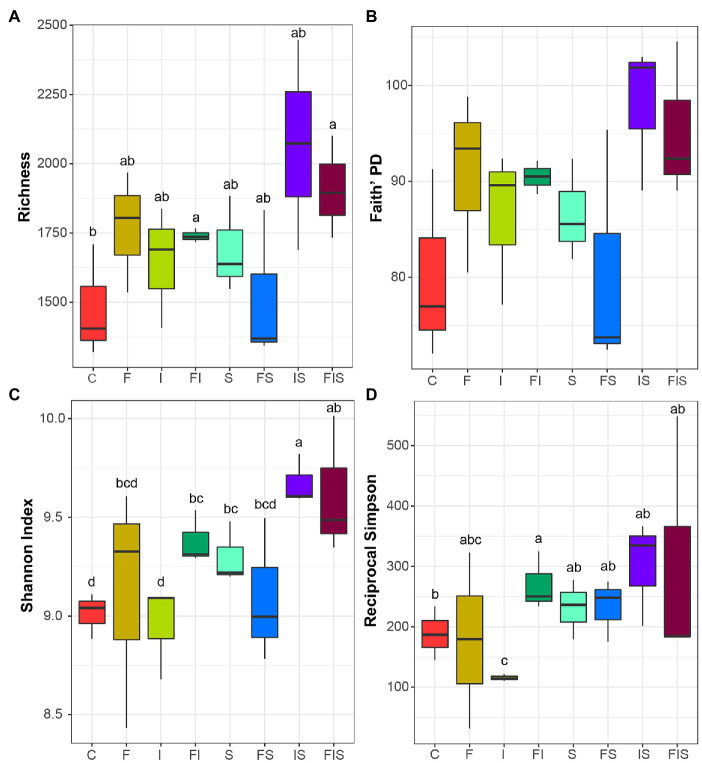
Alpha diversity boxplots from the rhizosphere microbial communities (order level) at the V15 stage: **(A)** total species richness, **(B)** Faith’s phylogenetic diversity, **(C)** Shannon index, and **(D)** Reciprocal Simpson index (1/D). Boxplots with the same letter or in absence are not significantly different (*p* < 0.05). Treatments: C, Control; F, Urea-topdressing fertilization at V5; I, *A. brasilense* inoculant on seeds sowing; FI, F + I; S, Maize stover coverage; FS, F + S; IS, I + S; FIS, F + I + S.

Rarefaction curves demonstrated sufficient sequencing coverage for each sample (Supplementary Figure 3). The number of amplicon sequence variants (ASVs) reached an asymptote for all treatments. Alpha diversity indices presented a considerable variability among treatment replicates with few significant differences (Figure 4), except for the comparisons (in general) against IS and FIS treatments. The abundance of ASVs was greater in IS and FIS than in C condition, and other treatments had intermediate diversity. This result shows that IS and FIS treatments tended to differ from the others.

**Figure 5:**
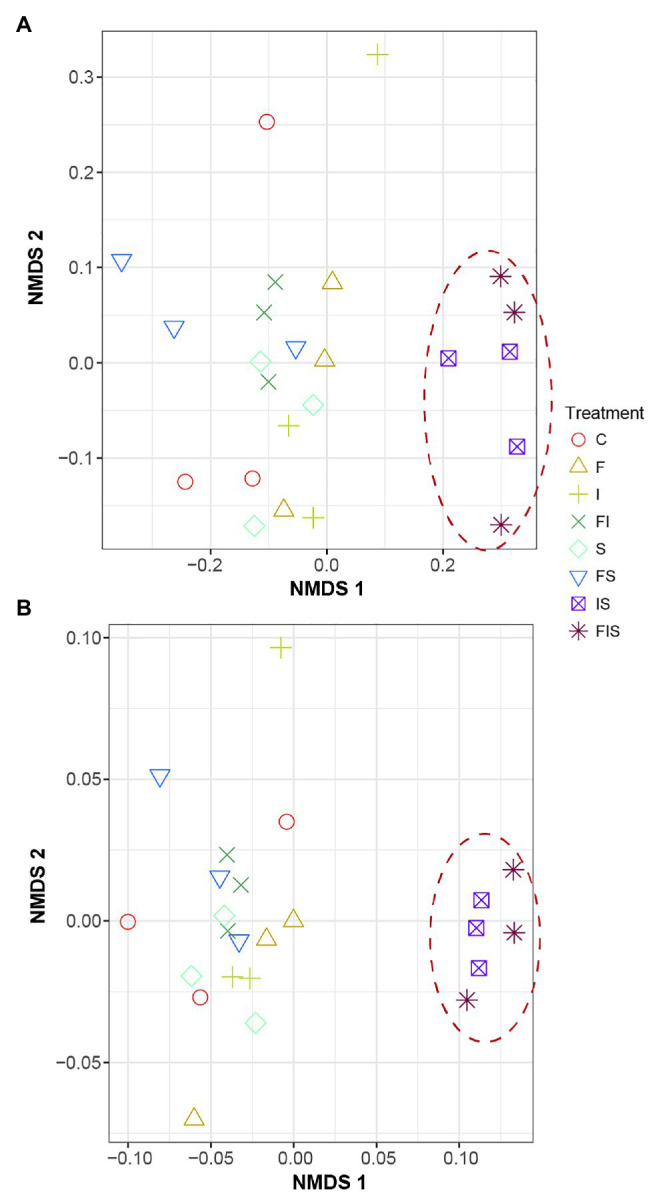
Non-metric multidimensional distance scaling (NMDS) analysis of the composition of the rhizosphere microbial communities among treatments at V15. **(A)** Taxonomic similarity (Bray-Curtis) and **(B)** phylogenetic similarity (weighted UniFrac). Treatments: C, Control; F, Urea-topdressing fertilization at V5; I, *A. brasilense* inoculant on seeds sowing; FI, F + I; S, Maize stover coverage; FS, F + S; IS, I + S; FIS, F + I + S.

Taxonomic (Bray-Curtis-R = 0.4157, *p* < 0.002) and phylogenetic (weighted UniFrac-R = 0.461, *p* < 0.001) approaches used to estimate community dissimilarities and beta diversity among rhizosphere-soil samples (Figure 5) demonstrated that IS and FIS were distinctly grouped from all other treatments.

### Structure of Microbial Communities

**Figure 6:**
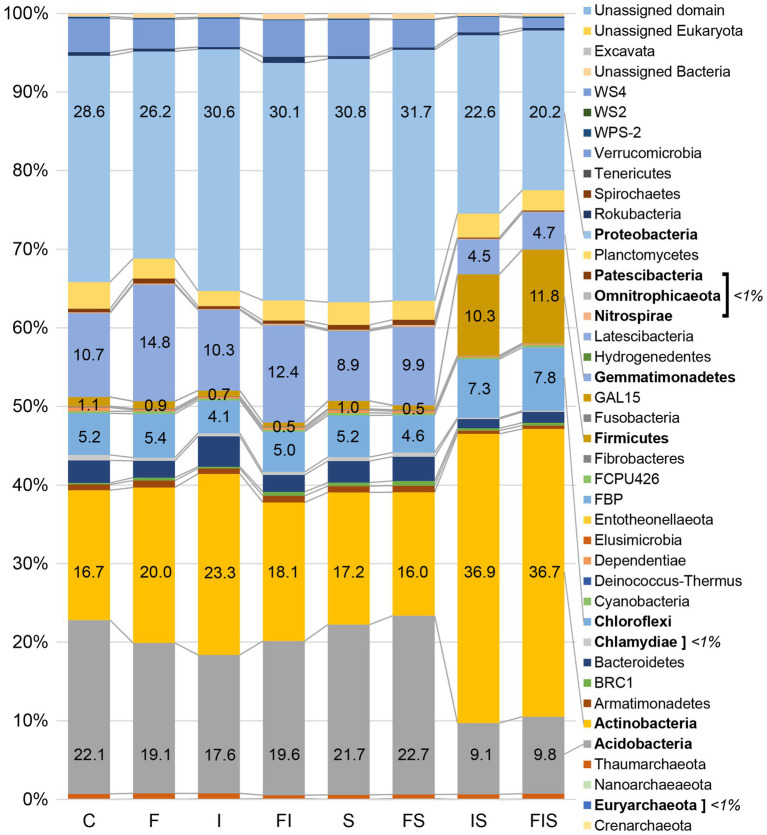
Relative abundances of the microbial communities at the phylum level from the 16S rRNA sequencing from the rhizosphere at V15 (*n* = 3). Sequences (*Q* > 30) were aligned against Silva 132 99% database using Qiime2 software platform version 2018.8 (https://qiime2.org/). Bars with percentages and names in bold are the most representative and significant phyla (*p* < 0.05). Treatments: C, Control; F, Urea-topdressing fertilization at V5; I, *A. brasilense* inoculant on seeds sowing; FI, F + I; S, Maize stover coverage; FS, F + S; IS, I + S; FIS, F + I + S.

A total of 10,497 ASVs were classified and distributed in 38 phyla, using Silva 132 99% database (Quast et al., 2013; Figure 6). The most abundant classes (>5% at least in one treatment), include Acidobacteriia, Actinobacteria, Alphaproteobacteria, Bacilli, Gammaproteobacteria, Gemmatimonadetes, and Thermoleophilia (Table 1), with significant increments of Actinobacteria (19.0%) and Bacilli (17.3%) in IS treatment, and Bacilli (9.7%) in FIS treatment.

**Table 1:** The most abundant classes in treatments (more than 5% in at least one treatment).

Class	Control (C)	Fertilizer (F)	Inoculant (I)	F + I (FI)	Stover (S)	F + S (FS)	I + S (IS)	F + I + S (FIS)
Acidimicrobiia	1.2%	1.7%	1.5%	1.7%	1.6%	1.8%	1.6%	1.6%
Acidobacteriia^∗^	16.1%	12.8%	11.7%	11.7%	15.7%	17.0%	5.1%	5.0%
Actinobacteria^∗^	5.2%	5.6%	10.9%	5.8%	6.0%	5.3%	19.0%	17.3%
Alphaproteobacteria^∗^	15.4%	13.8%	17.1%	15.7%	17.7%	17.8%	12.8%	10.9%
Bacilli^∗^	1.1%	0.9%	0.7%	0.5%	1.0%	0.5%	10.0%	9.7%
Bacteroidia^∗^	2.5%	1.9%	3.7%	2.0%	2.5%	2.8%	1.1%	1.0%
Blastocatellia (Subgroup 4)^∗^	1.6%	2.1%	1.9%	2.2%	1.5%	1.6%	0.9%	1.0%
Deltaproteobacteria^∗^	2.9%	3.5%	3.4%	3.7%	3.0%	2.8%	2.5%	2.6%
Gammaproteobacteria	10.5%	9.0%	10.3%	10.7%	10.2%	11.3%	7.4%	7.8%
Gemmatimonadetes^∗^	10.2%	14.5%	10.0%	12.1%	8.5%	9.4%	4.2%	3.7%
KD4-98^∗^	1.3%	1.0%	1.0%	1.6%	1.3%	1.1%	1.9%	2.1%
Ktedonobacteria	1.7%	2.0%	1.4%	1.3%	1.7%	1.2%	2.7%	2.4%
Planctomycetacia^∗^	2.7%	1.8%	1.5%	1.7%	2.2%	1.6%	2.6%	2.5%
Subgroup 6	3.7%	3.5%	3.2%	4.3%	3.3%	3.0%	2.6%	3.6%
Thermoleophilia	9.7%	12.1%	10.2%	9.4%	8.8%	8.2%	15.7%	18.0%
Verrucomicrobiae^∗^	4.3%	3.7%	3.6%	4.6%	4.6%	3.5%	2.0%	1.5%
Others	9.8%	10.1%	8.1%	10.8%	10.3%	10.9%	8.0%	9.3%

∗ Significant difference at *p* < 0.05.

**Figure 7:**
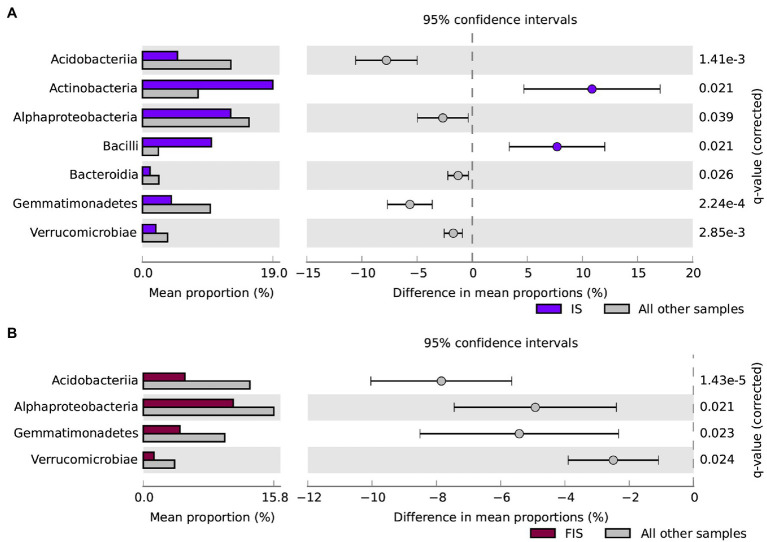
Statistical differences of representative microbial communities at the class level from the rhizosphere at V15. **(A)** *A. brasilense* inoculant at seed sowing + Maize stover coverage (IS) against all other treatments; **(B)** Urea-topdressing fertilization at V5 + *A. brasilense* inoculant at seed sowing + Maize stover coverage (FIS) against all other treatments. Classes overrepresented in IS and FIS (colored/dark) correspond to positive differences among proportions, and other samples (gray/light) correspond to negative differences among proportions. Error bars are the standard deviations. The *q*-values were calculated using the Storey FDR approach (*p* < 0.05) with effect size filter in difference among proportions (*DP* < 1.00).

At the class level, microbial taxa that significantly changed in abundance in IS compared to all other treatments, including Actinobacteria (19%), Alphaproteobacteria (12.8%), Bacilli (10%), Acidobacteriia (5.1%), Gemmatimonadetes (4.2%), Verrucomicrobiae (2%), and Bacteroidia (1.1%), with special attention to the increments of Actinobacteria and Bacilli in IS (Figure 7A). The FIS treatment had a significant reduction in Alphaproteobacteria (10.9%), Acidobacteriia (5%), Bacilli (11.5%), Gemmatimonadetes (3.7%), and Verrucomicrobia (1.5%) compared to all other treatments (Figure 7B).

## Discussion

Our results showed a high baseline effect promoted by the initial NPK fertilization in the furrow before seed sowing, which restricted our findings to each input effect. Even with this baseline, the effect of the inputs on treatments containing *A. brasilense* inoculant showed a reduction of soil CH_4_ emissions after V15 and before V5 to CO_2_ emissions. In addition, a tendency for plant biomass increases at V15 was also observed (maize plants are not fully grown at this stage, presenting early kernel development, which means only a prediction of plant biomass potential for the mature kernel stage). Such an effect is consistent with the expected benefit promoted by this PGPR, which can reduce nitrate and produce phytohormones like auxins – for example, indole-3-acetic acid (IAA; Bothe et al., 1992; Costacurta et al., 1994; Bashan et al., 2004), thus stimulating plants for a better root system development at initial stages and preparing them to an open-wide nutrient intake system for the subsequent reproductive stages (Vacheron et al., 2013; EMBRAPA, 2015).

Apparently, *A. brasilense* interactions with other microbes in rhizosphere communities respond to the maize stover addition and can reduce carbon-based GHG (CH_4_ and CO_2_) emissions. This was possibly the same phenomenon observed by Steinweg et al. (2008) as a consequence of carbon-use efficiency that has been increasing the soil carbon assimilation throughout the years (Allison et al., 2010). Despite the similar results of N_2_O among treatments, the growth promotion of the root system stimulated by *A. brasilense* (Costa et al., 2015) can contribute to nitrogen-use efficiency by plants from nitrogen inputs, removing nitrogen availability to denitrification pathways and allowing plant biomass gains as verified in-fields condition (Araújo et al., 2015). These carbon and nitrogen use efficiencies are correlated to a variation in the C:N ratio, serving as indicators of ecological stoichiometry, which can be used to monitor crop field conditions (Agren, 2004).

Although the presence of *A. brasilense* was not observed (data not shown) in our 16S rRNA amplicon sequencing data at the V15 sampling time, its benefits combined with the maize stover addition were determinant for biomass gains. In this case, the activation of the inoculation effects in our experiment occurred before V15, and *A. brasilense* may have been replaced by other microorganisms. In addition, Bonkowski et al. (2021) reported that the influence of plant growth stages is more significant than the fertilization level on the microbial community shift. Besides, the mature time of different roots and their rhizo deposits play an important role in microbiome assembly, which can cause the fluctuation in gene quantifications, as observed in our study.

On average, amendments of maize stover coverage since the beginning of the experiment (S treatments) stimulate a better development of maize plants (Figure 3) and can incite groups of microorganisms (Actinobacteria, Firmicutes, and Proteobacteria), allowing them to rise as a dominant later, as observed at V15 in the 16S rRNA amplicon sequencing profile. So, maize stover provides nutrients in favor of the bioaugmentation of these microbial groups as a prebiotic compound. On the other hand, maize stover decomposition seems to be primarily responsible for the increase in CO_2_ emissions during the entire experiment. However, these CO_2_ emissions from soils were reduced in the interaction of maize stover coverage with *A. brasilense* (IS treatment) among all combinations with stover coverage, mainly after V5. The increase of soil CO_2_ emissions could be an advantage for plant photosynthesis with the rapid availability of this gas for plant growth (Bond-Lamberty and Thomsom, 2010). Also, CO_2_ emissions might act as an offset to other GHG emissions due to the equilibrium of microbial activities (Liu and Greaver, 2009), as observed in our experiments with low levels of CH_4_ and N_2_O in comparison to CO_2_. Even though no significant differences were found in IS at V15 compared to the other treatments, the biomass of the plant could reach around 3.12 tons in-field conditions for IS, which means an increment of 0.6 tons and 0.34 tons compared to C and FI (the second in biomass gains at lower GHG emissions), respectively. It is worth mentioning that FIS was discarded from this in-field calculation for using more input with a small increment of 0.04 tons in comparison to IS. Small increments of each plant can improve biomass gains on a larger scale (60,000 plants ha^−1^), especially if the production has reached through low-cost management, such as the use of microbial inoculants (Santini et al., 2018).

Yang et al. (2017) also found a reduction in CO_2_ emissions (~4.5%) in treatments with biochar in comparison to maize stover, as amendments in maize crop. Biochar has a structural reordination of lignocellulose chains (after pyrolysis) that allows being colonized by beneficial groups of microorganisms (e.g., Proteobacteria, Actinobacteria, and Firmicutes) as found by Cannavan et al. (2016). However, Actinobacteria and Firmicutes were likely favored by the inoculation of *A. brasilense* and plant residues, increasing their abundance in IS and FIS treatments (Figure 6). Functionally, the maize rhizosphere actively selects groups from bulk soil that acts on carbon fixation and degradation, among other pathways, including exopolysaccharides and antibiotic production, to control soil-borne pathogens (Li et al., 2014). These functional groups are expected to be promoted since maize roots exudate, considering their production and composition, can largely support the microbiota demands (Carvalhais et al., 2011; Li et al., 2014). The favored bacterial groups that distinguished IS and FIS treatments from others (Figure 6) can act, indirectly, on the rhizosphere structure due to their influence as tenacious substrate competitors and antimicrobial producers. Besides, these groups have the ability of sporulation to survive in adverse conditions, such as drought. All these characteristics could make classes like Actinobacteria and Bacilli persistent in the environment (Figure 7A) – Actinobacteria are known as antibiotic producers, saprophytes, and PGPR (Doumbou et al., 2001), while Bacilli are PGPR, cellulose, and hemicellulose degraders, biosurfactants, and carotenoids producers, and act as biopesticides (Lee et al., 2008; Di Pasqua et al., 2014). However, the abundance of Acidobacteria dropped down in IS, an unexpected behavior of a ubiquitous and versatile class of microorganisms, which participate in carbohydrate and nitrogen metabolism (Kielak et al., 2016; Eichorst et al., 2018). All these functions need to be accessed using more detailed molecular approaches to evaluate these treatment interactions and identify the microorganisms at a more specific taxonomic level. Overall, the three factors evaluated separately (i.e., single-variable treatments) showed that: (a) urea-topdressing fertilization increased N_2_O emissions around 1-week after the application, as also reported by Calvo et al. (2016); (b) the inoculant, *A. brasilense* induced the reduction of CH_4_ and CO_2_ emissions; and (c) maize stover coverage showed an increment for CO_2_ emissions.

Following these patterns, when the inputs were combined, their effects were potentialized or merged: (a) the FI treatment showed intermediate values (between F and I treatments) of total CO_2_ and N_2_O emissions; (b) FS showed similar N_2_O and CO_2_ emissions in comparison to F and S treatments, respectively; (c) FIS merged patterns of GHG emissions from F, I, and S treatments; however, for CH_4_, FIS potentialized the responses of I and S for emission reduction, with the advantage of higher levels of biomass gains; and (d) the IS treatment showed similar positive responses to FIS, with the inoculant influence on the reduction of CO_2_ emissions, therefore rising as the best production treatment with low-cost input for higher biomass production and less GHG emissions. In conclusion, this study revealed the effects of input combinations on the maize soil-rhizosphere microbiota and GHG fluxes. The combination of microbial inoculant and maize stover coverage was found to be the best input option, aiming for high biomass production of maize plants with the beneficial reduction of CO_2_ equivalent emissions of the main GHGs. In addition, the microbial structure presented increments in the abundance of taxa related to carbon fixation, lignocelluloses degradation, and antibiotic production that might be responsible for GHG mitigation. Through our results related to the structure of the microbial communities, the stover coverage was pointed out as one of the responsible factors modulating microbial GHG fluxes, which should be evaluated using other more specific omics tools, such as metatranscriptomics and metaproteomics. Finally, it is worth mentioning that Glass and Orphan (2012) have tracked the composition of each enzyme related to CH_4_ and N_2_O pathways, indicating iron (Fe) and copper (Cu) as essential metal cofactors that might be explored in future research.

## Data Availability Statement

The original contributions presented in the study are publicly available. This data can be found at: The sequences are deposited in Sequence Read Archive (SRA) repository, the accession number is PRJNA495686, and the link is https://www.ncbi.nlm.nih.gov/bioproject/PRJNA495686/.

## Author Contributions

CY, LB, and ST conceived the project and designed the experiment. CY performed the greenhouse experiment. CY, AV, AF, and ML performed the molecular analyses. CY, LB, AV, and AF analyzed and contributed to interpreting the data. CY, LB, and AV wrote the manuscript. ST and JR critically reviewed the manuscript. All authors contributed to the article and approved the submitted version.

### Conflict of Interest

The authors declare that the research was conducted in the absence of any commercial or financial relationships that could be construed as a potential conflict of interest.

## Footnotes

^1^https://www.ipcc.ch/

^2^https://earthmicrobiome.org/
