# Supplementary material for: Responses of Low-Cost Input Combinations on the Microbial Structure of the Maize Rhizosphere for Greenhouse Gas Mitigation and Plant Biomass Production
Source: Front Plant Sci. 2021 Jun 30;12:683658. doi: 10.3389/fpls.2021.683658 (PMC8278312; doi:10.3389/fpls.2021.683658)
Supplement: Supplementary file 1 [file Data_Sheet_1.docx]

Supplementary Material

# Supplementary Figures

**
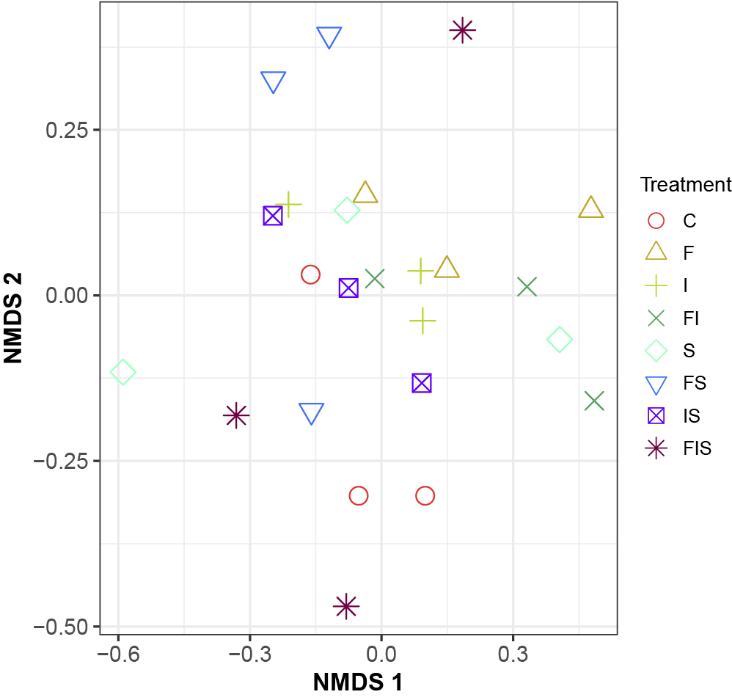
**

**Supplementary Figure 1.** Non-metric multidimensional distance scaling (NMDS) of the multivariate analysis of the soil physicochemical parameters among treatments. Treatments: C – Control; F – Urea-topdressing fertilization at V5; I – *A. brasilense* inoculant on seeds sowing; FI – F + I; S – Maize stover coverage; FS – F + S; IS – I + S; FIS – F + I + S.

**
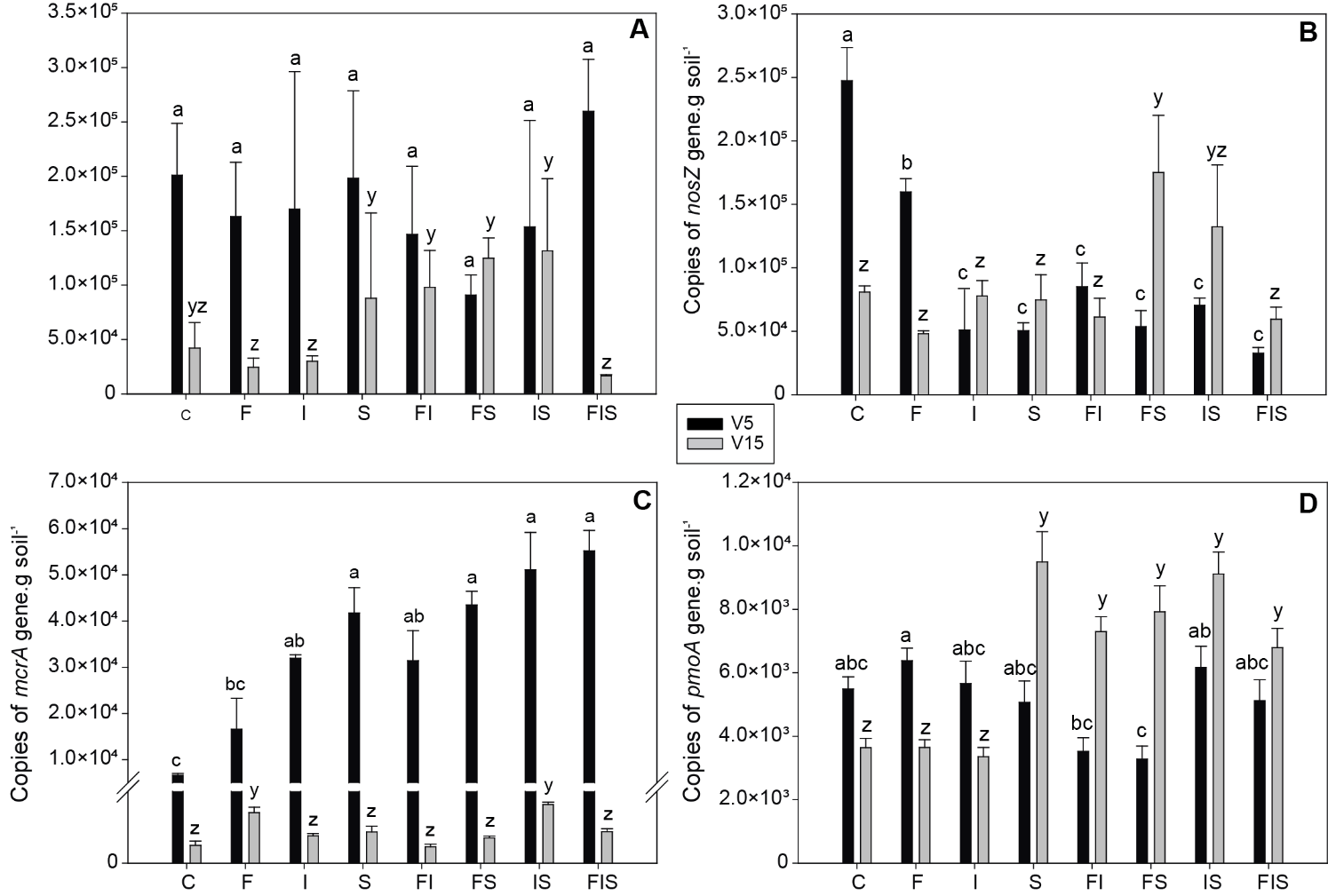
**

**Supplementary Figure 2.** Quantitative PCR of genes from the nitrogen – (A) *nifH* and (B) *nosZ* – and methane – (C) *mcrA* and (D) *pmoA* – cycles from the rhizosphere (n = 3; V5 and V15) for all factorial treatments. Bars with the same letter are not significantly different (*P* < 0.05). Two groups of series of letters compare treatments from distinctly stage periods (*abcd* for V5 and *xyz* for V15). Treatments: C – Control; F – Urea-topdressing fertilization at V5; I – *A. brasilense* inoculant on seeds sowing; FI – F + I; S – Maize stover coverage; FS – F + S; IS – I + S; FIS – F + I + S.

**
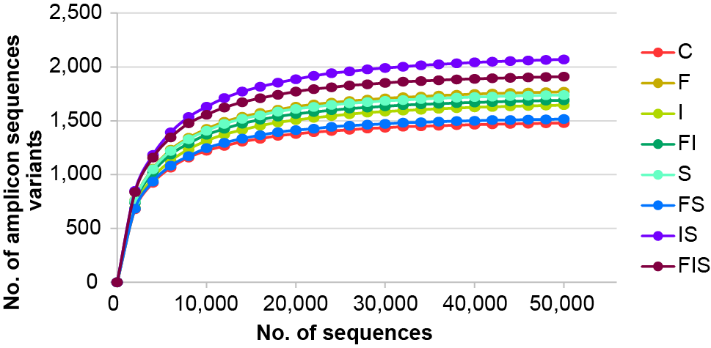
**

**Supplementary Figure 3.** Rarefaction curves of the total amplicon sequence variants (ASVs) obtained from all factorial treatments. Treatments: C – Control; F – Urea-topdressing fertilization at V5; I – *A. brasilense* inoculant on seeds sowing; FI – F + I; S – Maize stover coverage; FS – F + S; IS – I + S; FIS – F + I + S.

# Supplementary Tables

**Supplementary Table 1.** Soil chemical characteristics of the treatments at V15. Values are mean ± SE, n = 3. In each column, means followed by the same letter or letter absence are not significantly different (p < 0.05) based on ANOVA with Tukey HSD test and Kruskal-Wallis with Dunn test for normal and non-normal values, respectively. Ca, Mg, Al, potential acidity (H+Al), sum of bases (SB), and cation exchange capacity (CEC) are expressed in mmolc.kg^-1^. P is expressed in mg.kg^-1^. Organic matter (OM) is expressed in g.kg^-1^. Base saturation (V) and aluminum saturation (m) are expressed in %.

| **Treatment** | **pH** | **CEC** | **V** | **m** | **OM** | **P** | **K** | **Ca** | **Mg** | **Al** | **H+Al** | **SB** |
| --- | --- | --- | --- | --- | --- | --- | --- | --- | --- | --- | --- | --- |
| Control (C) | 6.2±0.1 a | 43.3±0.9 | 60.7±3.2 | <3.6±0.2 | 8.7±1.5 | 41.0±1.0 | 3.0±0.6 | 18.7±1.2 | 5.0±0.0 a | <1 | 16.7±1.2 | 26.7±1.8 |
| Fertilizer (F) | 6.2±0.1 a | 48.0±1.5 | 60.3±2.2 | <3.3±0.2 | 9.0±3.0 | 39.7±0.3 | 3.3±0.7 | 21.0±1.2 | 5.0±0.0 a | <1 | 18.7±0.9 | 29.3±1.8 |
| Inoculant (I) | 6.1±0.1 ab | 44.3±1.7 | 58.7±3.0 | <3.7±0.3 | 7.3±0.7 | 38.3±1.2 | 3.0±0.6 | 18.7±1.5 | 4.7±0.3 a | <1 | 18.0±0.6 | 26.3±2.2 |
| F + I (FI) | 6.1±0.1 ab | 43.0±2.9 | 56.3±4.1 | <4.0±0.5 | 8.3±1.2 | 37.0±0.6 | 2.3±0.3 | 17.3±2.6 | 5.0±0.6 a | <1 | 18.3±0.7 | 24.7±3.5 |
| Stover (S) | 6.3±0.1 a | 47.7±0.3 | 61.7±2.2 | <3.3±0.1 | 8.0±1.5 | 38.3±1.9 | 3.0±0.6 | 20.7±1.2 | 6.0±0.0 a | <1 | 18.0±1.0 | 29.7±1.2 |
| F + S (FS) | 5.6±0.1 b | 43.3±0.7 | 52.0±2.0 | <4.2±0.2 | 7.7±2.7 | 38.3±1.3 | 2.3±0.3 | 16.0±1.0 | 4.3±0.3 b | <1 | 20.7±0.9 | 22.7±1.2 |
| I + S (IS) | 6.1±0.1 ab | 43.3±0.3 | 56.3±3.2 | <3.9±0.2 | 8.7±0.7 | 37.7±0.7 | 2.3±0.9 | 17.3±0.7 | 5.0±0.0 a | <1 | 18.7±1.5 | 24.7±1.5 |
| F + I + S (FIS) | 6.1±0.1 ab | 42.0±3.1 | 60.7±2.3 | <3.8±0.2 | 7.7±0.3 | 36.7±1.2 | 3.7±2.2 | 16.7±0.7 | 5.3±0.3 a | <1 | 16.3±1.8 | 25.7±1.8 |

**Supplementary Table 2.** Primers used in the qPCR of nitrogen fixation (*nifH*), nitrous oxide reduction (*nosZ*), methanogenesis (*mcrA*), and methanotrophy (*pmoA*) genes with modified thermal cycling.

| **Gene** | **Primer** | | **Sequence** | | | | **Fragment length** | | **Reference** | | |
| --- | --- | --- | --- | --- | --- | --- | --- | --- | --- | --- | --- |
| *mcr*A | mlas-F (mod) | | 5’ GGYGGTGTMGGDTTCACMCARTA 3’ | | | | 469 | | Angel et al., 2012 | | |
|  | mcrA-R | | 5’ CGTTCATBGCGTAGTTVGGRTAGT 3’ | | | |  |  | Steinberg and Regan, 2009 | | |
| *pmo*A | A189F | | 5’ GGNGACTGGGACTTCTGG 3’ | | | | 472 | | Holmes et al., 1995 | | |
|  | mb661R | | 5’ CCGGMGCAACGTCYTTACC 3’ | | | |  |  | Costello and Lidstrom, 1999 | | |
| *nifH* | nifHF | | 5’ AAAGGYGGWATCGGYAARTCCACCAC 3’ | | | | 457 | | Rösch et al., 2002 | | |
|  | nifHR | | 5’ TTGTTSGCSGCRTACATSGCCATCAT 3’ | | | |  |  |  |  |  |
| *nos*Z | nosZ 2F | | 5’ CGCRACGGCAASAAGGTSMSSGT 3’ | | | | 267 | | Henry et al., 2006 | | |
|  | nosZ 2R | | 5’ CAKRTGCAKSGCRTGGCAGAA 3’ | | | |  |  |  |  |  |
| **Modified thermal cycling** | | | | | | | | | | | |
| **Gene** | **Initial**  **(10 min)** | **Cycles** | | **Denaturation**  **(30 s)** | **Annealing**  **(30 s)** | **Extension**  **(30 s)*** | | **Melting curve** | | | |
|  |  |  |  |  |  |  |  | **(15 s)** | | **(60 s)** | **(15 s)** |
| *mcr*A | 95 ºC | 50 cycles | | (95 ºC | 60 ºC | 72 ºC) | | 95 ºC | | 60 ºC | 95 ºC |
| *pmo*A |  |  |  |  | 58 ºC |  |  |  |  | 58 ºC |  |
| *nifH* |  |  |  |  | 59 ºC |  |  |  |  | 59 ºC |  |
| *nos*Z | 95 ºC | 6 cycles | | [95 ºC | 65 ºC (-1 ºC/cycle) | 72 ºC] | |  |  | 60 ºC |  |
|  |  | + 45 cycles | | (95 ºC | 60 ºC | 72 ºC) | |  |  |  |  |

*Acquisition data

**Supplementary Table 3.** Sequences of the primers used in the 16S amplicon sequencing based on the Earth Microbiome Project (http://www.earthmicrobiome.org/).

| **Sample** | **Type** | **Primer** |
| --- | --- | --- |
| forward | sequence structure | Illumina adapter + ATCTACAC + forward primer pad + forward primer link + **forward primer** |
| All | forward | 5’ AATGATACGGCGACCACCGAG ATCTACAC TATGGTAATT GT **GTGCCAGCMGCCGCGGTAA** 3’ |
| reverse | sequence structure | Reverse complement of 3’ Illumina adapter + Golay barcode + reverse primer pad + reverse primer link + **reverse primer** |
| 1C | reverse | 5' CAAGCAGAAGACGGCATACGAGAT AGCGGAGGTTAG AGTCAGTCAG CC **GGACTACHVGGGTWTCTAAT** 3' |
| 2C | reverse | 5' CAAGCAGAAGACGGCATACGAGAT ATCCTTTGGTTC AGTCAGTCAG CC **GGACTACHVGGGTWTCTAAT** 3' |
| 3C | reverse | 5' CAAGCAGAAGACGGCATACGAGAT TACAGCGCATAC AGTCAGTCAG CC **GGACTACHVGGGTWTCTAAT** 3' |
| 4F | reverse | 5' CAAGCAGAAGACGGCATACGAGAT ACCGGTATGTAC AGTCAGTCAG CC **GGACTACHVGGGTWTCTAAT** 3' |
| 5F | reverse | 5' CAAGCAGAAGACGGCATACGAGAT AATTGTGTCGGA AGTCAGTCAG CC **GGACTACHVGGGTWTCTAAT** 3' |
| 6F | reverse | 5' CAAGCAGAAGACGGCATACGAGAT TGCATACACTGG AGTCAGTCAG CC **GGACTACHVGGGTWTCTAAT** 3' |
| 7I | reverse | 5' CAAGCAGAAGACGGCATACGAGAT AGTCGAACGAGG AGTCAGTCAG CC **GGACTACHVGGGTWTCTAAT** 3' |
| 8I | reverse | 5' CAAGCAGAAGACGGCATACGAGAT ACCAGTGACTCA AGTCAGTCAG CC **GGACTACHVGGGTWTCTAAT** 3' |
| 9I | reverse | 5' CAAGCAGAAGACGGCATACGAGAT GAATACCAAGTC AGTCAGTCAG CC **GGACTACHVGGGTWTCTAAT** 3' |
| 10S | reverse | 5' CAAGCAGAAGACGGCATACGAGAT GTAGATCGTGTA AGTCAGTCAG CC **GGACTACHVGGGTWTCTAAT** 3' |
| 11S | reverse | 5' CAAGCAGAAGACGGCATACGAGAT TAACGTGTGTGC AGTCAGTCAG CC **GGACTACHVGGGTWTCTAAT** 3' |
| 12S | reverse | 5' CAAGCAGAAGACGGCATACGAGAT CATTATGGCGTG AGTCAGTCAG CC **GGACTACHVGGGTWTCTAAT** 3' |
| 13FI | reverse | 5' CAAGCAGAAGACGGCATACGAGAT CCAATACGCCTG AGTCAGTCAG CC **GGACTACHVGGGTWTCTAAT** 3' |
| 14FI | reverse | 5' CAAGCAGAAGACGGCATACGAGAT GATCTGCGATCC AGTCAGTCAG CC **GGACTACHVGGGTWTCTAAT** 3' |
| 15FI | reverse | 5' CAAGCAGAAGACGGCATACGAGAT CAGCTCATCAGC AGTCAGTCAG CC **GGACTACHVGGGTWTCTAAT** 3' |
| 16FS | reverse | 5' CAAGCAGAAGACGGCATACGAGAT CAAACAACAGCT AGTCAGTCAG CC **GGACTACHVGGGTWTCTAAT** 3' |
| 17FS | reverse | 5' CAAGCAGAAGACGGCATACGAGAT GCAACACCATCC AGTCAGTCAG CC **GGACTACHVGGGTWTCTAAT** 3' |
| 18FS | reverse | 5' CAAGCAGAAGACGGCATACGAGAT GCGATATATCGC AGTCAGTCAG CC **GGACTACHVGGGTWTCTAAT** 3' |
| 19IS | reverse | 5' CAAGCAGAAGACGGCATACGAGAT CGAGCAATCCTA AGTCAGTCAG CC **GGACTACHVGGGTWTCTAAT** 3' |
| 20IS | reverse | 5' CAAGCAGAAGACGGCATACGAGAT AGTCGTGCACAT AGTCAGTCAG CC **GGACTACHVGGGTWTCTAAT** 3' |
| 21IS | reverse | 5' CAAGCAGAAGACGGCATACGAGAT GTATCTGCGCGT AGTCAGTCAG CC **GGACTACHVGGGTWTCTAAT** 3' |
| 22FIS | reverse | 5' CAAGCAGAAGACGGCATACGAGAT AGTTACGAGCTA AGTCAGTCAG CC **GGACTACHVGGGTWTCTAAT** 3' |
| 23FIS | reverse | 5' CAAGCAGAAGACGGCATACGAGAT GCATATGCACTG AGTCAGTCAG CC **GGACTACHVGGGTWTCTAAT** 3' |
| 24FIS | reverse | 5' CAAGCAGAAGACGGCATACGAGAT CAACTCCCGTGA AGTCAGTCAG CC **GGACTACHVGGGTWTCTAAT** 3' |
